# Sex differences in clinical risk factors for Alzheimer's dementia patients with early-onset and late-onset

**DOI:** 10.3389/fgwh.2025.1601375

**Published:** 2025-08-04

**Authors:** Nathan Gerhard Faulstich, Sammy Hilmi Omar, Connor John O-brien, Dami Taiwo Ojo, Philip Cole Brewer, Emmanuel I. Nathaniel, Richard Goodwin, Laurie Roley, Adebobola Imeh-Nathaniel, Thomas I. Nathaniel

**Affiliations:** ^1^ University of South Carolina School of Medicine Greenville, Greenville, SC, United States; ^2^ Department of Biology, North Greenville University, Tigerville, SC, United States; ^3^ Department of Biomedical Engineering, University of South Carolina-Columbia, Columbia, SC, United States; ^4^ PRISMA Health, Upstate South Carolina, Greenville, SC, United States

**Keywords:** in Alzheimer's dementia, EAOD, LOAD risk factors, sex, male and female patients

## Abstract

**Background:**

The objective of this study is to identify the risk factors that contribute to sex differences in patients with Alzheimer dementia (AD), specifically focusing on Early-Onset Alzheimer's Dementia (EAOD) and Late-Onset Alzheimer Dementia (LOAD). Additionally, the study aims to determine whether these risk factors differ between male and female EAOD and LOAD patients.

**Methods:**

Our retrospective cohort study included a total of 6,212 patients diagnosed with either EOAD or LOAD from February 2016 to August 2020. Among this population, 687 patients (11.06%) were diagnosed with EOAD, while 5,525 patients (88.94%) had LOAD. We conducted a univariate analysis to identify differences in risk factors between male and female AD patients. A multivariate analysis was also performed to predict specific risk factors associated with male and female EOAD and LOAD patients.

**Results:**

In the adjusted analysis, males with LOAD were found to have significantly higher odds of several comorbidities, including dyslipidemia [Odds Ratio (OR) = 1.720, 95% Confidence Interval (CI) = 1.489–1.987, *p* < 0.001], peripheral vascular disease (OR = 2.324, 95% CI = 1.828–2.955, *p* < 0.001), obstructive sleep apnea (OR = 2.330, 95% CI = 1.768–3.070, *p* < 0.001), and pneumonia (OR = 1.235, 95% CI = 1.004–1.520, *p* = 0.046). In contrast, females with LOAD were associated with lower odds of having hypertension (OR = 0.715, 95% CI = 0.623–0.820, *p* < 0.001), osteoporosis (OR = 0.310, 95% CI = 0.254–0.380, *p* < 0.001), urinary tract infections (OR = 0.638, 95% CI = 0.521–0.782, *p* < 0.001), congestive heart failure (OR = 0.626, 95% CI = 0.481–0.815, *p* < 0.001), and rheumatoid arthritis. In male patients with EAOD the analysis indicated a strong association with gait dysfunction (OR = 10.797, 95% CI = 3.257–35.792, *p* < 0.001), peripheral vascular disease (OR = 3.835, 95% CI = 1.767–8.321, *p* < 0.001), and Chronic Obstructive Pulmonary Disease (COPD) (OR = 5.984, 95% CI = 2.186–16.381, *p* < 0.001). Conversely, females with EOAD were associated with significantly lower odds of experiencing cerebrovascular accidents (OR = 0.347, 95% CI = 0.155–0.778, *p* < 0.001), osteoporosis (OR = 0.345, 95% CI = 0.155–0.778, *p* = 0.030), and anxiety (OR = 0.412, 95% CI = 0.203–0.833, *p* = 0.014).

**Conclusions:**

Our findings indicate sex differences in the risk factors for EAOD and LOAD patients. Understanding these risk factors can help us develop strategies to improve diagnostic accuracy, create targeted interventions, and enhance clinical outcomes for both male and female EAOD and LOAD patients.

## Introduction

Alzheimer's dementia (AD) is a neurodegenerative disorder that profoundly affects various aspects of daily living. With the aging population expected to grow, the prevalence of AD is projected to triple in the coming decades, underscoring the urgent need to understand the complexities surrounding its causes and prevention (1). The clinical manifestations of AD may begin with early signs of language and visual changes, eventually progressing to characteristic symptoms such as memory loss, decline in language skills, behavioral changes, and impaired problem-solving abilities (2). Over time, these symptoms escalate, leading to severe complications and ultimately, death (3). Current insights into the disease's pathogenesis point to elevated levels of amyloid-beta (Aβ) proteins, which form extracellular plaques, and hyperphosphorylated tau proteins, which aggregate intracellularly to create neurofibrillary tangles (4).

AD remains the sixth leading cause of death in the United States and ranks as the fifth leading cause of death among Americans aged 65 and older, highlighting age as the primary risk factor for the disease (5, 6). EOAD refers to Alzheimer's that develops in individuals under the age of 65, while LOAD affects those aged 65 and older (2, 7, 8). Notably, LOAD impacts over 50% of individuals over the age of 85 and accounts for at least 80% of all Alzheimer's cases (9, 10). Risk factors associated with LOAD include sleep disorders, heart disease, diabetes, and obesity, among others (11, 12). Prominent symptoms of LOAD may consist of a progressive amnestic disorder characterized by episodic memory deficits, alongside varying degrees of executive, language, and visuospatial impairments (13, 14). As LOAD progresses, patients may experience physical decline, increased disability, and ultimately, death (2, 11).

EOAD represents approximately 10% of AD (15), with most cases being identified between the ages of 45 and 65 (16, 17). Symptoms of EAOD can vary considerably among individuals and may often mimic those of different forms of AD (16, 18). Early signs may include difficulties in retaining newly learned information or remembering significant dates, frequently asking for the same details, challenges in engaging in conversations or finding the appropriate words, misplacing items without recalling their location, and noticeable alterations in judgment, mood, and personality (16, 19). As the disease progresses, individuals may encounter severe mood swings and behavioral changes, profound confusion regarding time, place, and personal events, increasing suspicions toward friends, family, or caregivers, as well as difficulties in speaking, swallowing, or walking, all accompanied by significant memory loss (16).

The risk of developing AD is higher in females compared to males (7). One explanation for this disparity is that males tend to achieve higher levels of cognitive reserve than females (20). However, a recent study indicates that females present with higher cognitive reserve and resilience than males (21). Additionally, the cumulative impact of cognitive and social experiences on brain function and cognitive performance, which serves as a protective factor against AD, is reported to be more pronounced in males (22). Furthermore, females possess less cognitive reserve, resulting in a greater vulnerability to both LOAD and EOAD, along with a more rapid decline in cognitive functions (23).

In the AD population, females represent two-thirds of the cases and exhibit a higher risk of developing AD compared to males (24). The prevalence of individuals with AD is projected to rise more significantly among females than males in the coming years (25), a trend attributed to increased longevity in women as well as biological factors (26). In addition, the overall lifetime risk of acquiring AD for individuals aged 65 is 21.2% for females and 11.6% for males (25). While females have a higher likelihood of developing AD due to a combination of factors, including an extended life expectancy, hormonal fluctuations during menopause, and potential genetic susceptibility associated with the female sex chromosome (11, 25), our current research focuses on EOAD and LOAD. Given that more risk factors are linked to females than males within the typical AD population (27), we assume that males and females with EOAD and LOAD will exhibit differences in risk factors. We hypothesize that various factors—such as demographics, psychotropic medications including cholinesterase inhibitors (ChEIs), selective serotonin reuptake inhibitors (SSRIs), and second-generation antipsychotics (SGAs), along with medical history—may contribute to the observed sex differences in EOAD and LOAD patients. The current research effort is to contribute to continuing to better define sex differences in EOAD and LOAD patients for future investigation of the biological mechanisms of these differences in biological sex among EOAD and LOAD patients.

## Methods

### Study population

This study examined a cohort of AD patients treated at Prisma Health-Upstate (formerly known as Greenville Health System). Data were extracted from the Prisma Health-Upstate Alzheimer's Registry for patients treated between February 2016 and August 2020. The individuals included in this analysis had received a diagnosis of either EAOD or LOAD. Patients were classified as having AD using a combination of cognitive and neurological testing (MOCA, MMSE, etc.) and brain imaging (MRI, MRA, CT, CTA, etc.). Confirmation was performed using imaging and combination testing, including a p-tau 217 assay and a Beta Amyloid 42/40 ratio test. This is because beta-amyloid levels are one of the earliest markers present in the dementia disease process, and p-tau 217 is the most specific marker for AD. The study design was approved by Prisma Health's Institutional Review Board (IRB). Inclusion are confirmed cases of EOAD or LOAD, specifically those whose medical conditions were directly attributable to their neurological disorder. Patients with other neurological or psychiatric conditions, such as HIV encephalopathy or infectious encephalopathy, were excluded from this study.

Specific data extracted included demographics, medical history, medication use (including psychotropic agents), social risk factors, clinical risk factors, and laboratory values at admission for patients diagnosed with either EOAD or LOAD. The demographic variables analyzed included age, race, sex, and ethnicity. Race was classified into three categories: White, Black, and Other. Ethnicity was recorded as a binary variable, distinguishing between Hispanic and non-Hispanic patients. Additionally, data on various conditions and factors, such as anxiety, frailty, gait dysfunction, hallucinations, headaches, insomnia, mild cognitive impairment, obstructive sleep apnea, psychosis, secondary dementia, and traumatic head injury were collected.

Additionally, we collected data on various clinical risk factors, including arteriosclerosis, atrial fibrillation, unspecified cancer, cerebrovascular accidents, congestive heart failure, cold sores, chronic obstructive pulmonary disease (COPD), cutaneous ulcers, Down syndrome, ductal breast carcinoma, dyslipidemia, gastrointestinal (GI) ulceration, hypertension, insulin use, lung adenocarcinoma, small cell lung carcinoma, osteoporosis, pneumonia, peripheral vascular disease, rheumatoid arthritis, unspecified thyroid disease, upper respiratory infections, and urinary tract infections. The social risk factors analyzed encompassed histories of alcohol and tobacco use. Alcohol use was considered positive if a patient reported any alcohol consumption, regardless of the duration or quantity; this same criterion was applied to define positive tobacco use.

Data was extracted for medication history, including central acetylcholinesterase inhibitors (CAIs), second-generation antipsychotic agents (SGAs), selective serotonin reuptake inhibitors (SSRIs), and three additional medications that did not fit into the aforementioned categories. The CAIs comprised donepezil, galantamine, and rivastigmine. The SGAs included aripiprazole, olanzapine, and risperidone, while the SSRIs consisted of citalopram, escitalopram, and paroxetine. The variables for CAIs, SGAs, and SSRIs represented the count of patients taking at least one medication from each class. The specific medications listed subsequently indicate each frequency within its respective category. It is important to note that since some patients were on more than one medication within an individual class, the totals for CAIs, SGAs, and SSRIs may equal or fall short of the aggregate count of those specific medications. Furthermore, data on buspirone, memantine, and valproate usage was also collected. Unfortunately, data on dosages, frequencies, and therapy duration were unavailable for collection. Lastly, we extracted data on lab values for folate, vitamin B12, vitamin D, magnesium, calcium, thyroid-stimulating hormone (TSH), and homocysteine, which were recorded at the time of patient admission.

### Statistical analysis

Differences in demographics, pharmacological characteristics, social and clinical risk factors, and laboratory values were assessed using univariate statistical analysis. Data from patients with EAOD and LOAD were further stratified by sex. The Pearson *χ*^2^ test was used to evaluate nominal variables, while the t-test was used for the analysis of continuous variables. To avoid type 1 error inflation, we reduced the significance level (alpha) for each individual *t*-test. For example, if a typical alpha is 0.05, and multiple *t*-tests were run, the alpha level for each test is adjusted to 0.01. This provides the validity of our analysis, allowing us to control the probability of making at least one false positive error across all tests. We used adjusted the alpha level using the Bonferroni correction, where the alpha level is divided by the number of tests. Variables identified as statistically significant or nearing significance (*p*-value < 0.30) in the univariate analysis were used to construct a multivariable logistic regression model. Identifying variables “nearing significance” with a *p*-value < 0.30 indicates the broader threshold used in our analysis, allowing us to look at potential relationships that warrant further study with larger samples or different approaches. Adjusted analyses used the backward selection method based on the likelihood ratio. This approach was chosen to identify pertinent demographic, clinical, and pharmacologic risk factors that could be included in the model and removed if they did not contribute to its overall significance.

In each computed multivariable logistic regression model, the dependent variable was dichotomized into male or female, stratified by EOAD or LOAD. The independent variables included demographic, pharmacologic, clinical, and social factors, categorized according to onset (e.g., early vs. late). Logistic regression models and the corresponding odds ratios (OR) were considered to identify variables that were more likely to be associated with male vs. female patients for both EOAD and LOAD cohorts. A 95% confidence interval (CI) was considered for each independent variable in the final model. The calculated ORs were utilized to ascertain variables significantly associated with EOAD or LOAD. The models' sensitivity and specificity were assessed using the classification percentage and the area under the Receiver Operating Curve (AUROC). Multicollinearity among independent variables was examined using the Hosmer-Lemeshow test to determine associations between them. Cases exhibiting significant multicollinearity—where two variables demonstrated a statistically significant, strong correlation (*r* > 0.7)—were identified; in such instances, the variable with the lower OR was excluded from the model. This process was continued until all multicollinearity concerns were resolved. The flowchart for the variable selection process to identify risk factors in EOAD and LOAD and variables excluded from the models and stratified by sex are presented in Supplementary Figures S1 and S2. For all tests, a *p*-value of less than 0.05 was deemed significant. Analyses were conducted utilizing the Statistical Package for Social Sciences version 27.0 for Windows (SPSS, Chicago, IL).

## Results

In this study, a total of 6,212 patients diagnosed with EOAD or LOAD were identified. Among these patients, 687 were diagnosed with EOAD, representing 11.06% of the population, while 5,525 patients, or 88.94%, were diagnosed with LOAD (see Table 1). The demographic characteristics indicated that patients with EOAD were more likely to be female compared to males, with a distribution of 64.9% female to 35.1% male. A similar trend was observed in patients with LOAD, where 69.6% were female and 30.4% were male. Moreover, patients with LOAD were generally older, with a mean age of 86.31 years (±7.124), compared to the mean age of 74.84 years (±11.021) for patients with EOAD. Furthermore, patients diagnosed with EOAD demonstrated higher rates of certain conditions and behaviors. For example, 27.2% of EOAD patients reported alcohol use, compared to 15.2% of LOAD patients. Additionally, the prevalence of anxiety was higher in the EOAD group (11.9% vs. 9.3%), as well as rates of cancer (2.3% vs. 0.6%), Down syndrome (3.1% vs. 0.1%), hallucinations (1.5% vs. 0.6%), headaches (0.9% vs. 0.3%), and lung adenocarcinoma (2.3% vs. 0.1%). Patients with EAOD were more frequently prescribed cholinesterase inhibitors (ChEIs) (70.6% compared to 64.7%), second-generation antipsychotics (SGA) (19.8% vs. 13.1%), memantine (51.7% vs. 42.8%), and valproate (31.6% vs. 21.8%). In contrast, individuals with LOAD exhibited higher rates of certain conditions, including arteriosclerosis (0.6% vs. 0.0%), atrial fibrillation (8.7% vs. 5.5%), congestive heart failure (7.5% vs. 2.5%), hypertension (64.8% vs. 53.6%), insomnia (7.1% vs. 4.8%), osteoporosis (18.8% vs. 7.6%), upper respiratory infections (1.3% vs. 0.0%), and urinary tract infections (13.3% vs. 9.6%).

**Table 1:** Demographic and clinical characteristics of Alzheimer's dementia patients divided by early onset or late onset.

Characteristic	Early onset	Late onset	*P-*value
Number of patients	687	5,525	
Gender: no. (%)			
Male	241 (35.1)	1,680 (30.4)	0.012*^a^
Female	446 (64.9)	3,845 (69.6)	
Age group: no. (%)			
<50	3 (0.4)	0 (0)	<0.001*^a^
50–59	55 (8.0)	0 (0)	
60–69	151 (22.0)	46 (0.8)	
70–79	254 (37.0)	916 (16.6)	
≥80	224 (32.6)	4,563 (82.6)	
Mean ± SD	74.84 ± 11.021	86.31 ± 7.124	<0.001*^b^
Race: no. (%)			
Caucasians	573 (83.4)	4,595 (83.2)	0.168^a^
African Americans	94 (13.7)	679 (12.3)	
Other	7 (1.0)	148 (2.6)	
Hispanic ethnicity no. (%)	13 (1.9)	103 (1.9)	0.984^a^
Medical history: no. (%)			
Alcohol	186 (27.2)	830 (15.2)	<0.001*^a^
Anxiety	82 (11.9)	514 (9.3)	0.027*^a^
Arteriosclerosis	0 (0)	33 (0.6)	0.042*^a^
Atrial Fib	38 (5.5)	480 (8.7)	0.005*^a^
Cancer (unspecified)	16 (2.3)	31 (0.6)	<0.001*^a^
Cerebrovascular accident	56 (8.2)	399 (7.2)	0.378^a^
Congestive heart failure	17 (2.5)	413 (7.5)	<0.001*^a^
Cold sore	0 (0)	1 (0.0)	0.724^a^
COPD	9 (1.3)	42 (0.8)	0.132^a^
Cutaneous ulcer	0 (0)	4 (0.1)	0.481^a^
Down syndrome	21 (3.1)	8 (0.1)	<0.001*^a^
Ductal breast carcinoma	0 (0.0)	3 (0.1)	0.541^a^
Dyslipidemia	181 (26.3)	1,457 (26.4)	0.989^a^
Frailty	0 (0)	5 (0.1)	0.430^a^
Gait dysfunction	25 (3.6)	203 (3.7)	0.963^a^
GI ulceration	2 (0.3)	57 (1.0)	0.059^a^
Hypertension	368 (53.6)	3,578 (64.8)	<0.001*^a^
Hallucination	10 (1.5)	32 (0.6)	0.008*^a^
Headache (unspecified)	6 (0.9)	18 (0.3)	0.029*^a^
Insomnia	33 (4.8)	393 (7.1)	0.024*^a^
Insulin use	2 (0.3)	49 (0.9)	0.103^a^
Lung adenocarcinoma	16 (2.3)	4 (0.1)	<0.001*^a^
Small cell lung carcinoma	0 (0)	2 (0.0)	0.618^a^
Mild cognitive impairment	15 (2.2)	111 (2.0)	0.760^a^
Obstructive sleep apnea	38 (5.5)	288 (5.2)	0.724^a^
Osteoporosis	52 (7.6)	1,037 (18.8)	<0.001*^a^
Pneumonia	79 (11.5)	597 (10.6)	0.485^a^
Psychosis	1 (0.1)	28 (0.5)	0.190^a^
Peripheral vascular disease	41 (6.0)	395 (7.1)	0.253^a^
Rheumatoid arthritis	6 (0.9)	80 (1.4)	0.224^a^
Secondary dementia	9 (1.3)	42 (0.8)	0.132^a^
Tobacco	299 (43.5)	2,379 (43.1)	0.817^a^
Traumatic head Injury	2 (0.3)	34 (0.6)	0.291^a^
Thyroid disease (unspecified)	0 (0)	15 (0.3)	0.172^a^
Upper respiratory infection	0 (0)	71 (1.3)	0.003*^a^
Urinary tract infection	66 (9.6)	735 (13.3)	0.006*^a^
Medication history: no (%)			
Central acetylcholinesterase inhibitor (CAI)	485 (70.6)	3,572 (64.7)	0.002*^a^
Second generation antipsychotic (SGA)	136 (19.8)	725 (13.1)	<0.001*^a^
SSRI	215 (31.3)	1,844 (33.4)	0.275^a^
Buspirone	74 (10.8)	499 (9.0)	0.137^a^
Memantine	355 (51.7)	2,363 (42.8)	<0.001*^a^
Valproate	217 (31.6)	1,207 (21.8)	<0.001*^a^
Lab values: mean ± SD			
Folate	12.15 ± 4.35	12.28 ± 4.19	0.883^b^
B12	–	–	–
Vitamin D	30.03 ± 13.83	28.02 ± 14.94	0.651^b^
Magnesium	1.94 ± 0.29	1.95 ± 0.32	0.705^b^
Calcium	9.11 ± 0.67	9.06 ± 0.67	0.183^b^
TSH	2.96 ± 4.25	2.88 ± 5.94	0.889^b^
Homocystine	–	–	–

Results for continuous variables are presented as mean ± SD, while discrete data are presented as percentage frequency. Pearson's Chi-Square is used to compare differences between demographic and clinical characteristics in patients with early onset or late onset of AD.

^a^ Pearson's Chi-Squared test.

^b^ Student's *T* test.

*P-value < 0.05.

The comparison of risk factors for males and females with EAOD and LOAD is summarized in Table 2. Males with EAOD were significantly more likely to have a history of prior alcohol use (*p* < 0.001), dyslipidemia (*p* = 0.006), and tobacco use (*p* < 0.001). They also presented more frequently with gait dysfunction (*p* < 0.01), peripheral vascular disease (*p* < 0.01), mild cognitive impairment (*p* < 0.001), atrial fibrillation (*p* = 0.003), urinary tract infections (*p* = 0.005), congestive heart failure (*p* < 0.001), and chronic obstructive pulmonary disease (COPD) (*p* < 0.001). Additionally, males with EAOD were more likely to experience secondary dementia (*p* < 0.001), rheumatoid arthritis (*p* = 0.005), and Down syndrome (*p* = 0.031). They were also more likely to receive treatment with cholinesterase inhibitors (ChEIs) (*p* < 0.001) and memantine (*p* < 0.001). Furthermore, these individuals were more likely to have elevated levels of vitamin D (*p* < 0.001) and magnesium (*p* = 0.014).

**Table 2:** Demographic and clinical characteristics of male or female dementia patients stratified by early or late onset of dementia.

Characteristic	Early-onset		*P*-value	Late-onset		*P*-value
	Male	Female		Male	Female	
Number of patients	241	446		1,680	3,845	
Age group: no. (%)			<0.001*^a^			<0.001*^a^
<50 years	3 (1.2)	8,133 (16.0)		0 (0.0)	0 (0.0)	
50–59	25 (10.4)	3,956 (7.8)		0 (0.0)	0 (0.0)	
60–69	51 (21.2)	6,033 (11.9)		13 (0.8)	33 (0.9)	
70–79	103 (42.7)	11,047 (21.7)		273 (16.3)	643 (16.7)	
≥80	59 (24.5)	13,315 (26.2)		1,394 (83.0)	3,169 (82.4)	
Age mean ± SD	73.27 ± 10.716	75.69 ± 11.102	<0.001*^b^	85.69 ± 6.753	86.58 ± 7.265	<0.001*^b^
Race: no (%)			<0.001*^a^			<0.001*^a^
Caucasians	196 (81.3)	377 (84.5)		1,440 (85.7)	3,155 (82.1)	
African Americans	34 (14.1)	60 (13.5)		172 (10.2)	507 (13.2)	
Other	11 (4.5)	9 (2.0)		68 (4.2)	183 (4.8)	
Hispanic ethnicity: no. (%)	8 (3.3)	5 (1.1)	0.078^a^	15 (0.9)	95 (2.5)	<0.001*^a^
Medical history: no. (%)						
Alcohol use (%)	32.9	24.0	0.013*^a^	20.5	12.9	<0.001*^a^
Tobacco use (%)	63.9	32.5	<0.001*^a^	63.6	34.1	<0.001*^a^
Insomnia	10 (4.1)	23 (5.2)	0.556^a^	119 (7.1)	274 (7.1)	0.955^a^
Hypertension	123 (51.0)	245 (54.9)	0.329^a^	1,054 (62.7)	2,524 (65.6)	0.038*^a^
Dyslipidemia	68 (28.2)	113 (25.3)	0.414^a^	574 (34.2)	883 (23.0)	<0.001*^a^
Cerebrovascular accident	11 (4.6)	45 (10.1)	0.012*^a^	139 (8.3)	260 (6.8)	0.046*^a^
Osteoporosis	7 (2.9)	45 (10.1)	<0.001*^a^	145 (8.6)	892 (23.2)	<0.001*^a^
Gait dysfunction	20 (8.3)	5 (1.1)	<0.001*^a^	67 (4.0)	136 (3.5)	0.412^a^
Peripheral vascular disease	27 (11.2)	14 (3.1)	<0.001*^a^	192 (11.4)	203 (5.3)	<0.001*^a^
Atrial Fib	17 (7.1)	21 (4.7)	0.199^a^	146 (8.7)	334 (8.7)	0.996^a^
Hallucination	0 (0)	10 (2.2)	0.019*^a^	4 (0.2)	28 (0.7)	0.027*^a^
Cancer (unspecified)	0 (0.0)	16 (3.6)	0.003*^a^	13 (0.8)	18 (0.5)	0.162^a^
Anxiety	18 (7.5)	64 (14.3)	0.008*^a^	145 (8.6)	369 (9.6)	0.256^a^
UTI	27 (11.2)	39 (8.7)	0.297^a^	167 (9.9)	568 (14.8)	<0.001*^a^
URI	0	0	N/A	26 (1.5)	45 (1.2)	0.252^a^
Thyroid disease (unspec)	0	0	N/A	1 (0.1)	14 (0.4)	0.045*^a^
Insulin use	0 (0)	2 (0.4)	0.298^a^	12 (0.7)	37 (1.0)	0.366^a^
Secondary dementia	7 (2.9)	2 (0.4)	0.007*^a^	14 (0.8)	28 (0.7)	0.679^a^
Cold sore	0	0	N/A	0 (0.0)	1 (0.0)	0.509^a^
Frailty	0	0	N/A	1 (0.1)	4 (0.1)	0.613^a^
GI ulceration	0 (0.0)	2 (0.4)	0.298^a^	12 (0.7)	45 (1.2)	0.123^a^
Lung adenocarcinoma	0 (0.)	16 (3.6)	0.003*^a^	0 (0.0)	4 (0.1)	0.186^a^
Small cell carcinoma of lung	0 (0.0)	0 (0.0)	N/A	6 (0.4)	6 (0.2)	0.140^a^
Squamous cell ca of lung	0	0	N/A	2 (0.0)	0 (0.0)	0.032*^a^
Large cell carcinoma of lung	0 (0.0)	0 (0.0)	N/A	0 (0.0)	0 (0.0)	N/A
Mild cognitive impairment	13 (5.4)	2 (0.4)	<0.001*^a^	10 (0.6)	101 (2.6)	<0.001*^a^
Headache (unspec)	1 (0.4)	5 (1.1)	0.342^a^	0 (0.0)	18 (0.5)	0.005*^a^
Congestive heart failure	15 (6.2)	2 (0.4)	<0.001*^a^	104 (6.2)	309 (8.0)	0.016*^a^
Obstructive sleep apnea	18 (7.5)	20 (4.5)	0.102*^a^	140 (8.3)	148 (3.8)	<0.001*^a^
Arteriosclerosis	0	0	N/A	33 (2.0)	0 (0.0)	<0.001*^a^
Cutaneous ulcer	0	0	N/A	0 (0.0)	4 (0.0)	0.186^a^
Psychosis	1 (0.4)	0 (0.0)	0.173^a^	14 (0.8)	14 (0.4)	0.024*^a^
COPD	26 (10.8)	9 (2.0)	<0.001*^a^	139 (8.3)	424 (11.0)	0.002*^a^
Traumatic head injury	0 (0.0)	2 (0.4)	0.298^a^	3 (0.2)	31 (0.8)	0.006*^a^
Vasospasm	0 (0.0)	0 (0.0)	N/A	0 (0.0)	0 (0.0)	N/A
Pneumonia	24 (10.0)	55 (12.3)	0.352^a^	188 (11.2)	399 (10.4)	0.367^a^
Rheumatoid arthritis	4 (1.7)	2 (0.4)	0.103^a^	6 (0.4)	74 (1.9)	<0.001*^a^
Ductal carcinoma of breast	0 (0.0)	0 (0.0)	N/A	0 (0.0)	3 (0.0)	0.252^a^
Subclavian artery disease	0 (0.0)	0 (0.0)	N/A	0 (0.0)	0 (0.0)	N/A
Teratoma	0 (0.0)	0 (0.0)	N/A	0 (0.0)	0 (0.0)	N/A
Microangiopathy	0 (0.0)	0 (0.0)	N/A	0 (0.0)	0 (0.0)	N/A
Down syndrome	12 (5.0)	9 (2.0)	0.031*^a^	8 (0.5)	0 (0.0)	<0.001*^a^
Gastric erosion	0 (0.0)	0 (0.0)	N/A	0 (0.0)	0 (0.0)	N/A
Senile tremor	0 (0.0)	0 (0.0)	N/A	0 (0.0)	0 (0.0)	N/A
Medications no. (%)						
Central AchEI	199 (82.6)	286 (64.1)	<0.001*^a^	1,114 (66.3)	2,458 (63.9)	0.080^a^
SGA	28 (11.6)	108 (24.2)	<0.001*^a^	242 (14.4)	483 (12.6)	0.062^a^
SSRI	68 (28.2)	147 (33.0)	0.201^a^	451 (26.8)	1,393 (36.2)	<0.001*^a^
Memantine	146 (60.6)	209 (46.9)	<0.001*^a^	801 (47.7)	1,562 (40.6)	<0.001*^a^
Buspirone	22 (9.1)	52 (11.7)	0.307^a^	156 (9.3)	343 (8.9)	0.663^a^
Valproate	35 (14.5)	182 (40.8)	<0.001*^a^	389 (23.2)	818 (21.3)	0.120^a^
Lab values: mean ± SD						
Folate (B9)	11.108 ± 3.1123	13.192 ± 5.2403	0.036*^b^	12.618 ± 4.1157	12.100 ± 4.2305	0.568^b^
Cobalamin (B12)	No data	No data	N/A	No data	No data	N/A
Vitamin D	30.483 ± 11.2487	29.583 ± 17.1376	0.268^b^	25.151 ± 11.9906	29.324 ± 15.6465	0.014*^b^
TSH	2.08600 ± 1.943749	3.55711 ± 5.205862	0.005*^b^	2.30905 ± 1.888944	3.08459 ± 6.822133	0.002*^b^
Magnesium	2.001 ± 0.2764	1.889 ± 0.2996	0.745^b^	1.948 ± 0.2983	1.945 ± 0.3245	0.069^b^
Serum calcium	9.023 ± 0.6369	9.153 ± 0.6820	0.265^b^	8.976 ± 0.6345	9.099 ± 0.6755	0.054^b^

Results for continuous variables are presented as mean ± SD, while discrete data are presented as percentage frequency. Pearson's Chi-Square is used to compare differences between demographic and clinical characteristics in male or female AD patients.

^a^ Pearson's Chi-Squared test.

^b^ Student's *T* test.

*P-value < 0.05.

Females with EAOD were generally older (*p* < 0.001) and showed a higher likelihood of experiencing various health issues, including cerebrovascular accidents (*p* < 0.001), osteoporosis (*p* < 0.001), hallucinations (*p* < 0.001), cancer (*p* < 0.001), anxiety (*p* < 0.001), gastrointestinal ulceration (*p* = 0.005), lung cancer (*p* < 0.001), headaches (*p* = 0.005), insulin use (*p* < 0.005), traumatic head injury (*p* = 0.005), pneumonia (*p* = 0.006), and rheumatoid arthritis (*p* = 0.001). Additionally, these females were more likely to be treated with cholinesterase inhibitors (ChEIs), second-generation antipsychotics (SGAs), memantine, and valproate (*p* < 0.001). Furthermore, they exhibited higher levels of serum folate (*p* < 0.001) and thyroid-stimulating hormone (TSH) (*p* = 0.005).

In patients with LOAD, males were more likely to have a history of alcohol and tobacco use (*p* < 0.001). They also had a higher prevalence of several medical conditions, including arteriosclerosis (*p* < 0.001), dyslipidemia (*p* < 0.001), cerebrovascular accidents (*p* < 0.001), gait dysfunction (*p* = 0.007), peripheral vascular disease (PVD) (*p* < 0.001), cancer (*p* = 0.003), secondary dementia (*p* = 0.013), small cell carcinoma (*p* = 0.002), obstructive sleep apnea (*p* < 0.001), psychosis (*p* = 0.001), pneumonia (*p* = 0.007), and Down syndrome (*p* < 0.001). Additionally, these patients were more likely to be treated with medications such as memantine (*p* < 0.001), buspirone (*p* = 0.012), and valproate (*p* < 0.001). They also presented with elevated levels of vitamin B9 (*p* = 0.010) and magnesium (*p* < 0.001).

In contrast, females with LOAD were older (*p* < 0.001) and more frequently identified as Black or Hispanic (*p* < 0.001). They were also more likely to have a medical history that included osteoporosis (*p* < 0.001), atrial fibrillation (*p* = 0.019), hallucinations (*p* < 0.001), anxiety (*p* = 0.004), urinary tract infections (*p* < 0.001), upper respiratory infections (*p* = 0.004), thyroid disease (*p* = 0.001), insulin therapy (*p* = 0.007), cold sores (*p* = 0.009), frailty (*p* = 0.001), gastrointestinal ulceration (*p* = 0.002), lung adenocarcinoma (*p* = 0.003), mild cognitive impairment (*p* < 0.001), headaches (*p* = 0.001), congestive heart failure (*p* = 0.016), chronic obstructive pulmonary disease (COPD) (*p* = 0.001), head injury (*p* = 0.001), rheumatoid arthritis (*p* < 0.001), ductal breast cancer (*p* = 0.004), hypertension (*p* < 0.001), and migraines (*p* < 0.001). Furthermore, females were more likely to be prescribed selective serotonin reuptake inhibitors (SSRIs) (*p* < 0.001). They also tended to have higher serum levels of vitamin D (*p* = 0.014), calcium (*p* < 0.001), and thyroid-stimulating hormone (TSH) (*p* < 0.001).

**Figure 1:**
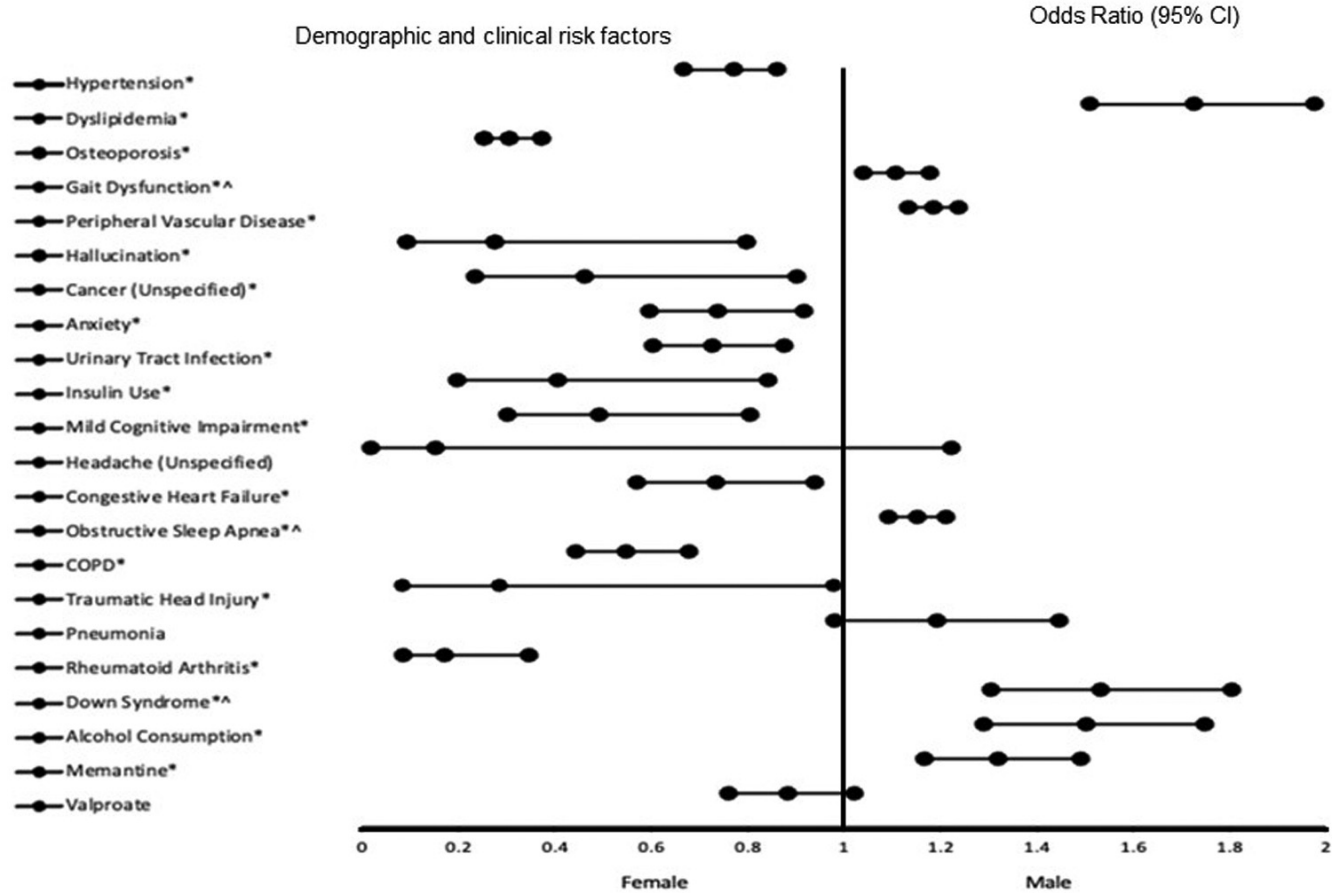
The forest plot illustrates the risk factors of EAOD and LOAD in the total population. Adjusted odds ratios (ORs) less than 1 indicate associations with being female, while adjusted ORs greater than 1 indicate associations with being male. Asterisks (*) denote statistically significant results (*P* < 0.05) with a 95% confidence interval. Caret symbols (^) indicate variables transformed by taking the fifth square root. The logistic regression model demonstrated strong predictive ability, with an area under the curve (AUC) of 0.747 (95% CI: 0.734–0.761, *P* < 0.001).

The results of the adjusted analysis exploring risk factors associated with EAOD and LOAD in the total population are presented in Figure 1. As illustrated in the figure, male patients with EOAD or LOAD exhibited significant associations with several factors, including: - dyslipidemia [Odds Ratio (OR) = 1.726, 95% Confidence Interval (CI), 0.666–0.860, *p* < 0.001], gait dysfunction (OR = 1.663, 95% CI, 1.212–2.281, *p* = 0.002), peripheral vascular disease (OR = 2.331, 95% CI, 1.865–2.914, *p* < 0.001) and obstructive sleep apnea (OR = 2.011, 95% CI, 1.552–2.606, *p* < 0.001). Others factors are down syndrome (OR = 8.478, 95% CI, 3.765–19.089, *p* < 0.001), alcohol use (OR = 1.502, 95% CI, 1.289–1.750, *p* < 0.001), and treatment with memantine (OR = 1.320, 95% CI, 1.168–1.491, *p* < 0.001) In contrast, female patients demonstrated associations with hypertension (OR = 0.757, 95% CI, 0.666–0.860, *p* < 0.001, osteoporosis (OR = 0.307, 95% CI, 0.252–0.374, *p* < 0.001), hallucinations (OR = 0.274, 95% CI, 0.094–0.796, *p* = 0.017), cancer (OR = 0.460, 95% CI, 0.234–0.903, *p* = 0.024), anxiety (OR = 0.739, 95% CI, 0.596–0.916, *p* = 0.006), urinary tract infections (OR = 0.726, 95% CI, 0.602–0.877, *p* < 0.001), insulin therapy (OR = 0.407, 95% CI, 0.196–0.844, *p* = 0.016), mild cognitive impairment (OR = 0.493, 95% CI, 0.301–0.805, *p* = 0.005) and congestive heart failure (OR = 0.733, 95% CI, 0.571–0.940, *p* = 0.015). Other factors are chronic obstructive pulmonary disease (COPD) (OR = 0.549, 95% CI, 0.443–0.680, *p* < 0.001), traumatic head injury (OR = 0.287, 95% CI, 0.084–0.981, *p* = 0.046), and rheumatoid arthritis (OR = 0.172, 95% CI, 0.086–0.345, *p* < 0.001) The predictive power of the logistic regression model is strong, as indicated by an area under the curve (AUC) of 0.747 (95% CI, 0.734–0.761, *p* < 0.001).

**Figure 2:**
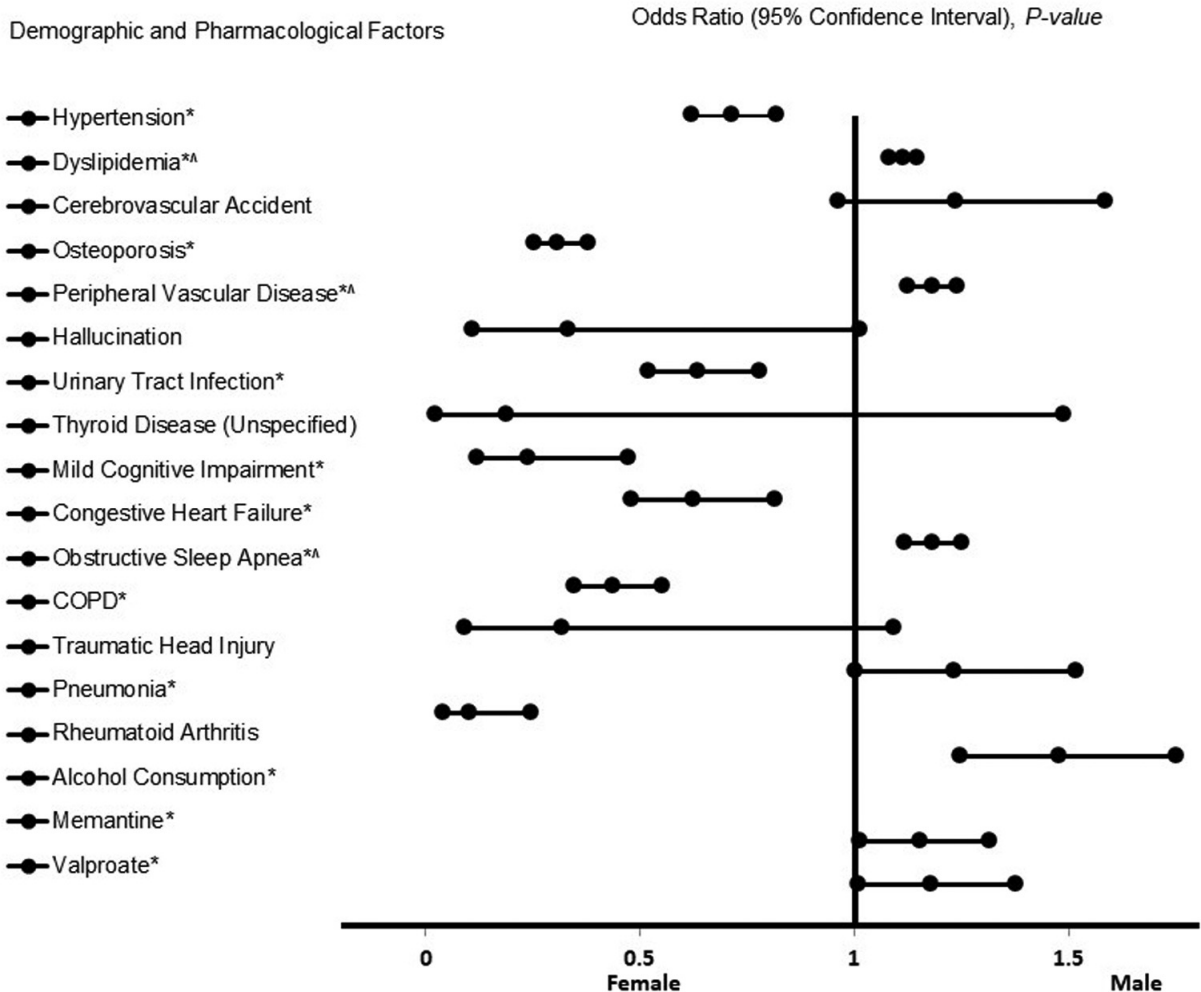
The forest plot represents risk factors of LOAD. Adjusted odds ratios (OR) less than 1 indicate associations with female sex, while ORs greater than 1 indicate associations with male sex. Asterisks (*) indicate statistical significance (*P* < 0.05) with a 95% confidence interval. Carets (^) denote transformed variables using the fifth square root. Model diagnostics showed an acceptable fit, with a Hosmer–Lemeshow test *P* < 0.001 and a Cox & Snell *R*^2^ of 0.156. The logistic regression model achieved an overall classification accuracy of 69.6%.

The results of the adjusted analysis examining risk factors associated with males vs. females in patients with LOAD are presented in Figure 2. The figure presents the forest plot representation of risk factors associated with LOAD. As shown in the figure, males with LOAD showed significant associations with several factors, including dyslipidemia (OR = 1.720, 95% CI, 1.489–1.987, *p* < 0.001), peripheral vascular disease (OR = 2.324, 95% CI, 1.828–2.955, *p* < 0.001), obstructive sleep apnea (OR = 2.330, 95% CI, 1.768–3.070, *p* < 0.001), pneumonia (OR = 1.235, 95% CI, 1.004–1.520, *p* = 0.046), alcohol consumption (OR = 1.480, 95% CI, 1.250–1.753, *p* < 0.001), including the use of memantine (OR = 1.156, 95% CI, 1.014–1.318, *p* = 0.030) and valproate (OR = 1.180, 95% CI, 1.010–1.379, *p* = 0.037). In contrast, females with LOAD were associated with hypertension (OR = 0.715, 95% CI, 0.623–0.820, *p* < 0.001), osteoporosis (OR = 0.310, 95% CI, 0.254–0.380, *p* < 0.001), urinary tract infections (OR = 0.638, 95% CI, 0.521–0.782, *p* < 0.001), mild cognitive impairment (OR = 0.240, 95% CI, 0.122–0.475, *p* < 0.001), congestive heart failure (OR = 0.626, 95% CI, 0.481–0.815, *p* < 0.001), chronic obstructive pulmonary disease (COPD) (OR = 0.439, 95% CI, 0.349–0.553, *p* < 0.001), and rheumatoid arthritis (OR = 0.104, 95% CI, 0.043–0.248, *p* < 0.001). Our model exhibited a strong predictive power for stroke (AUC = 0.747, 95% CI, 0.743–0.760, *p* < 0.001).

**Figure 3:**
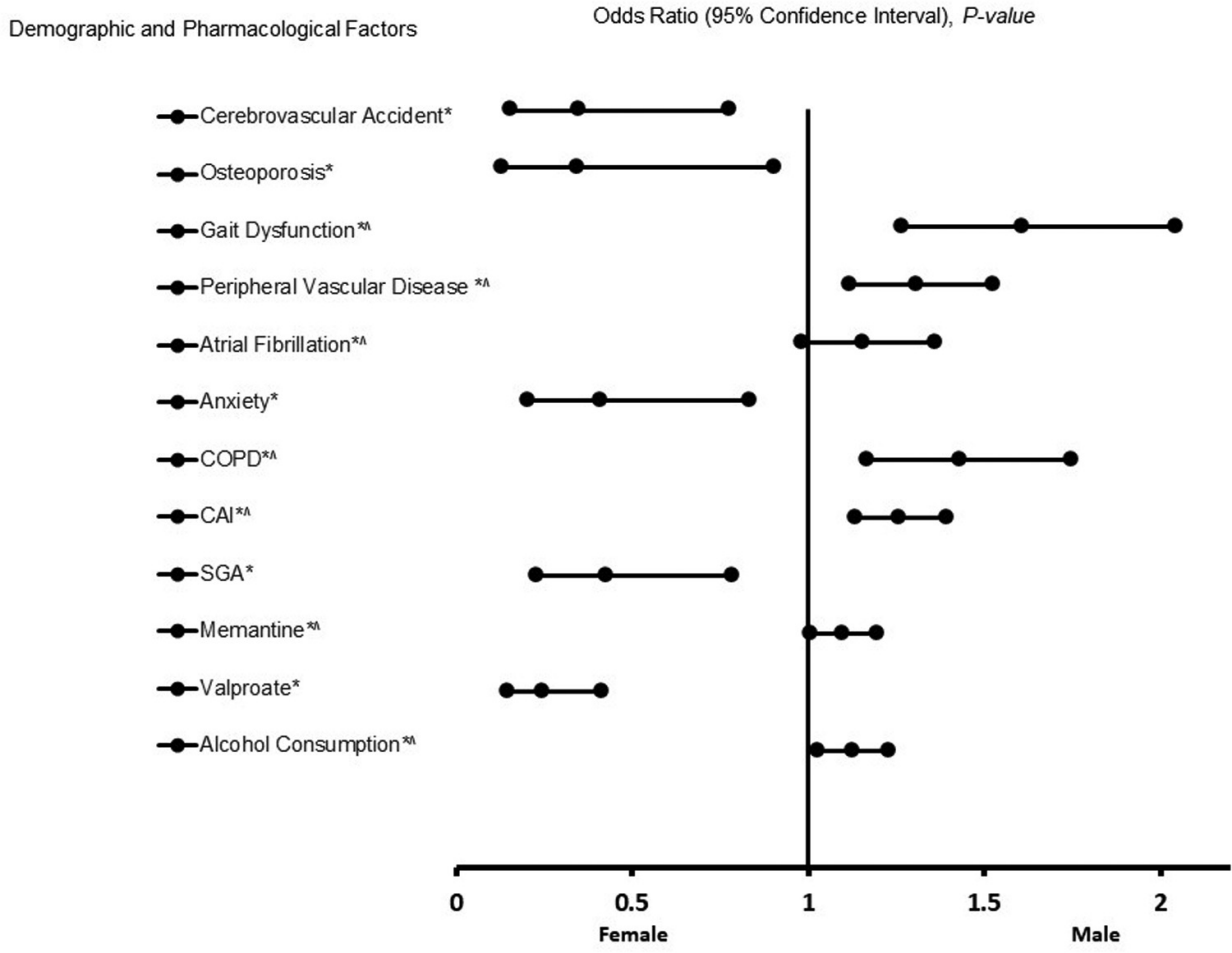
The forest plot displays risk factors of EAOD. An adjusted odds ratio (OR) less than 1 indicates an association with female sex, whereas an adjusted OR greater than 1 indicates an association with male sex. Asterisks (*) denote statistical significance (*P* < 0.05) with a 95% confidence interval. Caret symbols (^) indicate that the data were transformed by taking the fifth square root. Model diagnostics showed good explanatory power and fit, with a Hosmer–Lemeshow goodness-of-fit test yielding *P* < 0.001 and a Cox & Snell *R*^2^ of 0.319. The logistic regression model correctly classified 78.7% of cases overall.

The results of the adjusted analysis comparing males and females among patients with EOAD are presented in Figure 3. The figure presents the forest plot representation of risk factors associated with EOAD. As shown in the figure, male patients with EOAD were associated with gait dysfunction [Odds Ratio (OR) = 10.797, 95%, CI, 3.257–35.792, *p* < 0.001], peripheral vascular disease (OR = 3.835, 95% CI, 1.767–8.321, *p* < 0.001), Chronic Obstructive Pulmonary Disease (COPD) (OR = 5.984, 95% CI, 2.186–16.381, *p* < 0.001), the use of cholinesterase inhibitors (ChEIs) (OR = 3.141, 95% CI, 1.881–5.244, *p* < 0.001), memantine (OR = 1.587, 95% CI, 1.029–2.447, *p* = 0.037), and alcohol use (OR = 1.801, 95% CI, 1.155–2.809, *p* = 0.010). In contrast, female patients with EOAD were associated with cerebrovascular accidents (OR = 0.347, 95% CI, 0.155–0.778, *p* < 0.001), osteoporosis (OR = 0.345, 95% CI, 0.155–0.778, *p* = 0.030), anxiety (OR = 0.412, 95% CI, 0.203–0.833, *p* = 0.014), the use of second-generation antipsychotic agents (SGA) (OR = 0.425, 95% CI, 0.230–0.786, *p* = 0.006), and valproate (OR = 0.246, 95% CI, 0.146–0.414, *p* < 0.001). The results from the logistic regression indicate robust predictive power [Area Under the Curve (AUC) = 0.834, 95% CI, 0.801–0.866, *p* < 0.001].

In summary, our results (Table 3) reveal differences and similarities in risk factors associated with male and female patients. Males with LOAD exhibit vascular and metabolic comorbidities, respiratory issues, and alcohol use, and are more frequently treated with memantine and valproate. In contrast, females with LOAD tend to present with cardiovascular, inflammatory, and infectious conditions, along with osteoporosis**.**In EOAD, males are primarily associated with gait dysfunction, vascular and respiratory conditions, and a history of alcohol use, receiving cholinesterase inhibitors and memantine more often. Females with EOAD show links to cerebrovascular events and osteoporosis, with greater use of second-generation antipsychotics (SGAs) and valproate. Across both LOAD and EOAD, vascular and respiratory conditions are more prevalent in males, while osteoporosis and valproate use are recurring themes in females. These patterns highlight the importance of sex-specific considerations in the clinical management of EOAD and LOAD patients.

**Table 3:** The observed patterns stratified by sex based on LOAD and EAOD.

Category	Males	Females	Shared/notable patterns
LOAD (Late-Onset AD)	- Vascular/metabolic (dyslipidemia, PVD) - Respiratory (sleep apnea, pneumonia) - Alcohol use - Treated with memantine, valproate	- Cardiovascular/inflammatory (HTN, CHF, RA) - Cognitive & infectious (UTI, MCI) - Osteoporosis - COPD	- Gender-specific comorbidities - Osteoporosis appears in both LOAD and EOAD in females
EOAD (Early-Onset AD)	- Gait dysfunction - Vascular/respiratory (PVD, COPD) - Alcohol use - Treated with ChEIs, memantine	- Cerebrovascular accidents - Osteoporosis - Treated with SGAs, valproate	- Memantine use in both LOAD/EOAD males - Valproate use in both LOAD/EOAD females

## Discussion

Early-onset dementia, which is diagnosed before the age of 65, shares some risk factors with LOAD. However, the differences in risk factors between males and females with LOAD and EOAD are not yet fully understood. This study explores the differences in risk factors based on sex in patients diagnosed with LOAD and EOAD. We observed that 11.6% of our population comprises EAOD while 88.94% comprises LOAD. This finding is not surprising, as only a small proportion of people with AD experience EAOD, typically representing around 5%–10% of cases. In contrast, the majority (90%–95%) experience LOAD (16).

Our results showed that males with LOAD were linked to several health issues, including dyslipidemia, peripheral vascular disease, obstructive sleep apnea, pneumonia, alcohol use, and the use of medications such as memantine and valproate. In contrast, females with LOAD were associated with conditions such as hypertension, osteoporosis, urinary tract infections, mild cognitive impairment, congestive heart failure, chronic obstructive pulmonary disease (COPD), and rheumatoid arthritis. For EAOD, males were associated with gait dysfunction, peripheral vascular disease, COPD, and the use of cholinesterase inhibitors (ChEIs), memantine, and alcohol use. Conversely, female patients with EAOD had associations with cerebrovascular accidents, osteoporosis, anxiety, and treatment using valproate.

Other studies have reported similar results for males with LOAD for dyslipidemia, which is known to exacerbate cognitive decline and increase the risk of AD (12, 28). Additionally, peripheral vascular disease contributes to mixed dementia (29), while obstructive sleep apnea increases cognitive decline, worsening existing AD (30).Pneumonia is another concern, as it can lead to pneumonia-facilitated mortality in AD patients (31). Treatments such as cholinesterase inhibitors (ChEIs) (32), memantine (33), and valproate (32) been shown in other studies to address various symptoms of AD.

Our findings related to male EAOD patients are also supported by another study that associates gait dysfunction with AD (33). Individuals with EAOD often exhibit slower gait speed, increased step variability, and difficulties turning, even before significant cognitive decline becomes evident (34). Other risk factors, including peripheral vascular disease (30), chronic obstructive pulmonary disease (COPD), and alcohol consumption (35) have been reported by other studies to be associated with cognitive decline in AD patients (31). Furthermore, pneumonia has been linked to pneumonia-facilitated mortality in patients with AD (31).

Our results indicate that male LOAD and EAOD are both associated with PVD and alcohol use, suggesting a shared underlying pathology linked to vascular dysfunction and alcohol consumption in these conditions. The connection between LOAD and EOAD with PVD implies that individuals suffering from PVD may face an increased risk of developing both forms of Alzheimer's due to common risk factors, such as compromised blood circulation affecting both the brain and extremities.

In general, vascular dysfunction in AD significantly impacts brain metabolism, homeostasis, and the clearance of β-amyloid and other toxic metabolites (36). Vascular factors play a vital role at multiple stages of the pathogenesis of LOAD (37) and EOAD (36), often preceding the onset of classical symptoms, gross pathological changes, and cognitive impairments associated with these conditions. Alterations in vessel hemodynamics, angiogenesis, vascular cell function, vascular coverage, blood-brain barrier permeability, and immune cell migration may be interconnected with amyloid toxicity, oxidative stress, and the apolipoprotein E (APOE) genotype (38). These vascular deficiencies may subsequently contribute to amyloid deposition in the brain, neurotoxicity, glial activation, and metabolic dysfunction across various cell types. This may create a vicious feedback loop, leading to progressively worsening neuronal and vascular pathology throughout the progression of LOAD and EOAD. Future therapeutic strategies should focus on addressing vascular dysfunction and inflammation at both the EOAD and LOAD stages of Alzheimer's pathogenesis or even in pre-symptomatic individuals.

We observed that alcohol use was associated with male LOAD and EOAD patients**.** Alcohol use has been identified as the most substantial modifiable risk factor for the onset of dementia (39). While abstaining from alcohol is linked to a reduced risk of mortality compared to unmanaged alcohol use disorders, the risk of developing AD remains unchanged following abstinence (39). Furthermore, alcohol use is associated with various other independent risk factors for AD, making it a key contributor to all types of dementia (40). Notably, there is a significant link between alcohol use and an increased risk of AD in male patients, with evidence suggesting that heavy consumption can accelerate disease progression and elevate the likelihood of developing AD (41). Collectively, existing studies support our findings of an association between alcohol use and male patients with LOAD and EOAD, implying that alcohol consumption may considerably impact these patients, potentially exacerbating their condition or complicating their care. Future longitudinal research could further explore the specific relationship between alcohol consumption and male patients with LOAD and EOAD.

Our observation that both male LOAD and EOAD are treated with memantine suggests that therapeutic interventions currently available can effectively address both conditions. Furthermore, while male LOAD patients received treatment with a cognitive enhancing agent (ChEIs), male EOAD patients were prescribed valproate, highlighting differences in the psychotropic agents utilized for treating male patients with LOAD and EOAD.

Our finding reveals that female patients with LOAD were associated with several risk factors, including osteoporosis, which elevates the risk of falls and fractures in older individuals with AD (42). Additionally, we identified hypertension, which is known to contribute to AD (43), and urinary infections that exacerbate AD symptoms in patients (44). Moreover, mild cognitive impairment has been reported as a precursor to AD (45). At the same time, congestive heart failure can lead to reduced cerebral blood flow, impairing cognitive function and contributing to dementia symptoms (46). Chronic Obstructive Pulmonary Disease (COPD) is also associated with an increased risk of cognitive impairment and AD, particularly in those with significant declines in lung function (47). Furthermore, rheumatoid arthritis can lead to inflammation that diminishes blood flow to essential organs in AD patients (48).

Our findings regarding female patients with EOAD align with previous research showing that cerebrovascular accidents harm brain tissue by disrupting blood flow, resulting in cognitive decline and impaired functioning that can lead to AD (49), and osteoporosis (42). Anxiety is often an early indicator of AD, especially in the initial stages of the disease, and may even precede noticeable cognitive deterioration (50). We also observed that females diagnosed with EOAD were more often treated with valproate. Evidence indicates that valproate has neuroprotective properties pertinent to AD (51). Through various signaling pathways, valproate can promote the neurogenesis of neural progenitor and stem cells both *in vitro* and *in vivo* (52). While valproate is frequently utilized to manage aggression, agitation, and behavioral disturbances in AD (53), current data suggest that adverse effects are more common among individuals taking this medication (54, 55). Nonetheless, the potential benefits may outweigh the risks in managing female patients with EOAD.

An important finding from our current study is the association between osteoporosis, EOAD, and LOAD in female patients. Generally, individuals with osteoporosis face a higher risk of developing dementia compared to those without the condition (56). Osteoporosis is recognized as an early risk factor for AD (57). Furthermore, a substantial connection has been reported between osteoporosis and female patients with AD, highlighting that osteoporosis not only correlates with AD but that the two conditions also exhibit a bidirectional relationship (58). This suggests that females diagnosed with EOAD and LOAD are at an elevated risk of developing osteoporosis, primarily due to hormonal changes associated with menopause, which significantly affect bone density and increase fracture risk (59). Additionally, fractures themselves serve as an independent risk factor for AD (60). Patients suffering from both osteoporosis and AD face heightened risks for morbidity and mortality (42), and those affected by either EOAD or LOAD alongside osteoporosis or fractures may endure even more severe consequences. There is a need for future large-scale studies to investigate further the relationship between osteoporosis-related dementia, particularly in female patients with EOAD and LOAD.

Understanding sex-specific risk factors in AD including both EOAD and LOAD subtypes, is of critical clinical and scientific importance**.** Emerging evidence suggests that biological sex influences the manifestation, progression, and treatment response in AD. Recognition of sex-specific risk profiles can facilitate earlier diagnosis, improve risk stratification, and inform more effective prevention strategies. Furthermore, clinical approaches that account for sex-related variables—such as hormonal differences, comorbidities, and medication responses—may enhance individualized patient care. Integrating these considerations into both research and practice is essential for advancing diagnostic precision and therapeutic efficacy. Finally, acknowledging and addressing sex-based differences in EOAD and LOAD can lead to more personalized and equitable care for individuals affected by this neurodegenerative disease.

## Conclusion

Despite significant advancements in research examining sex differences in specific risk factors affecting treatment outcomes for both female and male AD patients, notable gaps still exist. Current data frequently fail to identify risk factors that contribute to sex differences in EOAD and LOAD patients. Our findings revealed both differences and similarities in risk factors between male and female patients. Specifically, we identified unique risk factors for each sex that, when addressed, could improve the care of EOAD and LOAD patients. Our results underscore the need for developing management strategies that target the specific risk factors contributing to sex differences in EOAD and LOAD patients.

## Limitations

This retrospective data analysis was from a single institution, so the data cannot be generalized to other institutions and populations. Additionally, electronic medical records were used for the data analysis, allowing for the possibility of human error to limit the efficacy of the results. The database did not provide information about the consumption rate for alcohol and tobacco, and thresholds to define people as users. Also, there is a lack of sufficient healthy control samples to compare against identified risk factors, especially when analyzing these factors by sex. In addition, the database did not provide data about the progression of risk factor exposure before diagnosis. Moreover, dosage, duration, or adherence data were not unavailable. In addition, there is lack of information on whether the EOAD cases are linked to known AD causative mutations. Despite these limitations, the findings are relevant and compelling. They can be generalized to a substantial cohort of individuals experiencing LOAD and EAOD, given the high prevalence of identified risk factors among both male and female participants within this study.

## Data Availability

The datasets presented in this study can be found in online repositories. The names of the repository/repositories and accession number(s) can be found in the article/Supplementary Material.
