# Supplementary material for: Sex differences in clinical risk factors for Alzheimer's dementia patients with early-onset and late-onset
Source: Front Glob Womens Health. 2025 Aug 4;6:1601375. doi: 10.3389/fgwh.2025.1601375 (PMC12358484; doi:10.3389/fgwh.2025.1601375)
Supplement: Supplementary file 1 [file Datasheet1.docx]

Supplement Figure 1. The flowchart for the Variable Selection Process to Identify Risk Factors in EOAD and LOAD and variables excluded from the model.


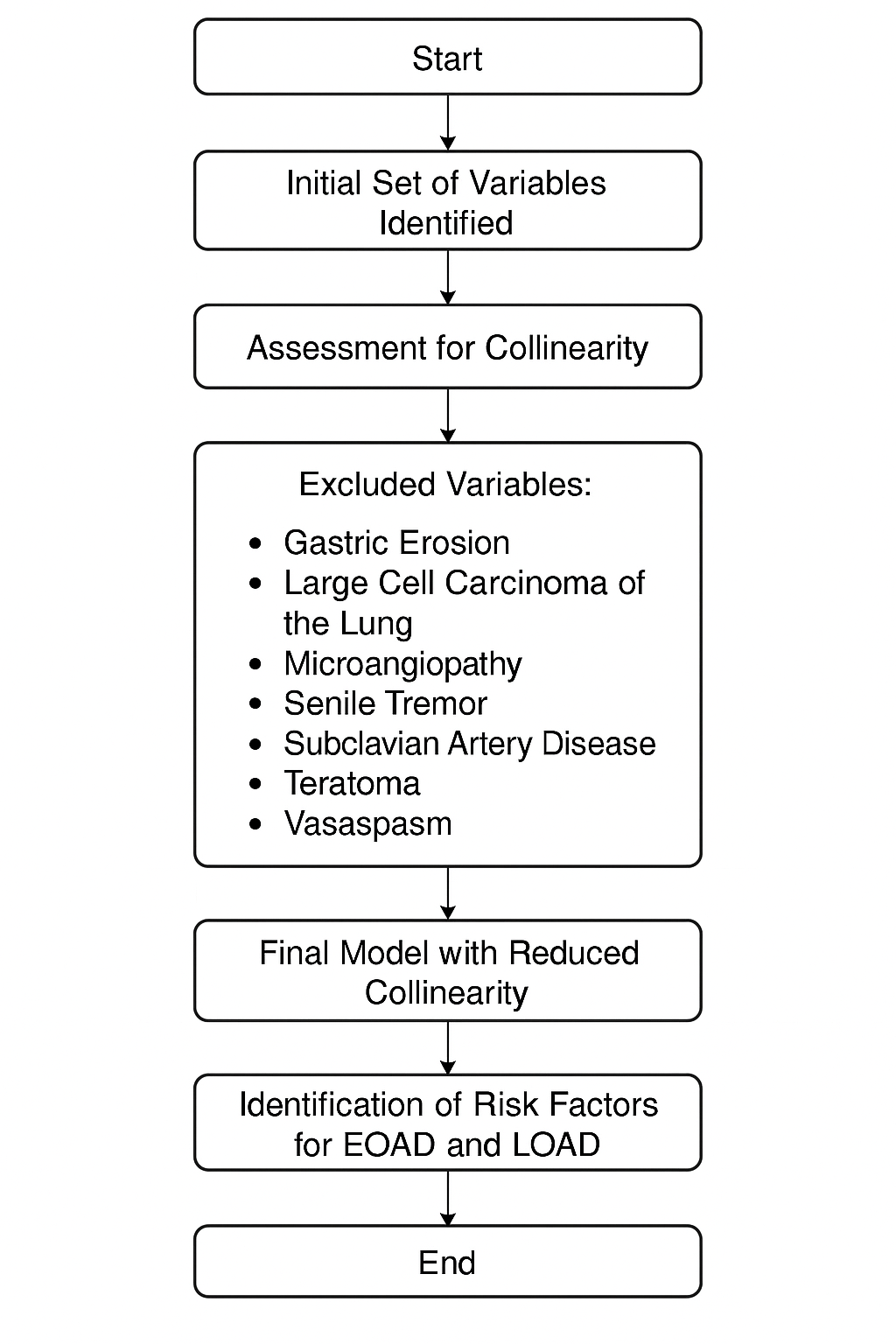


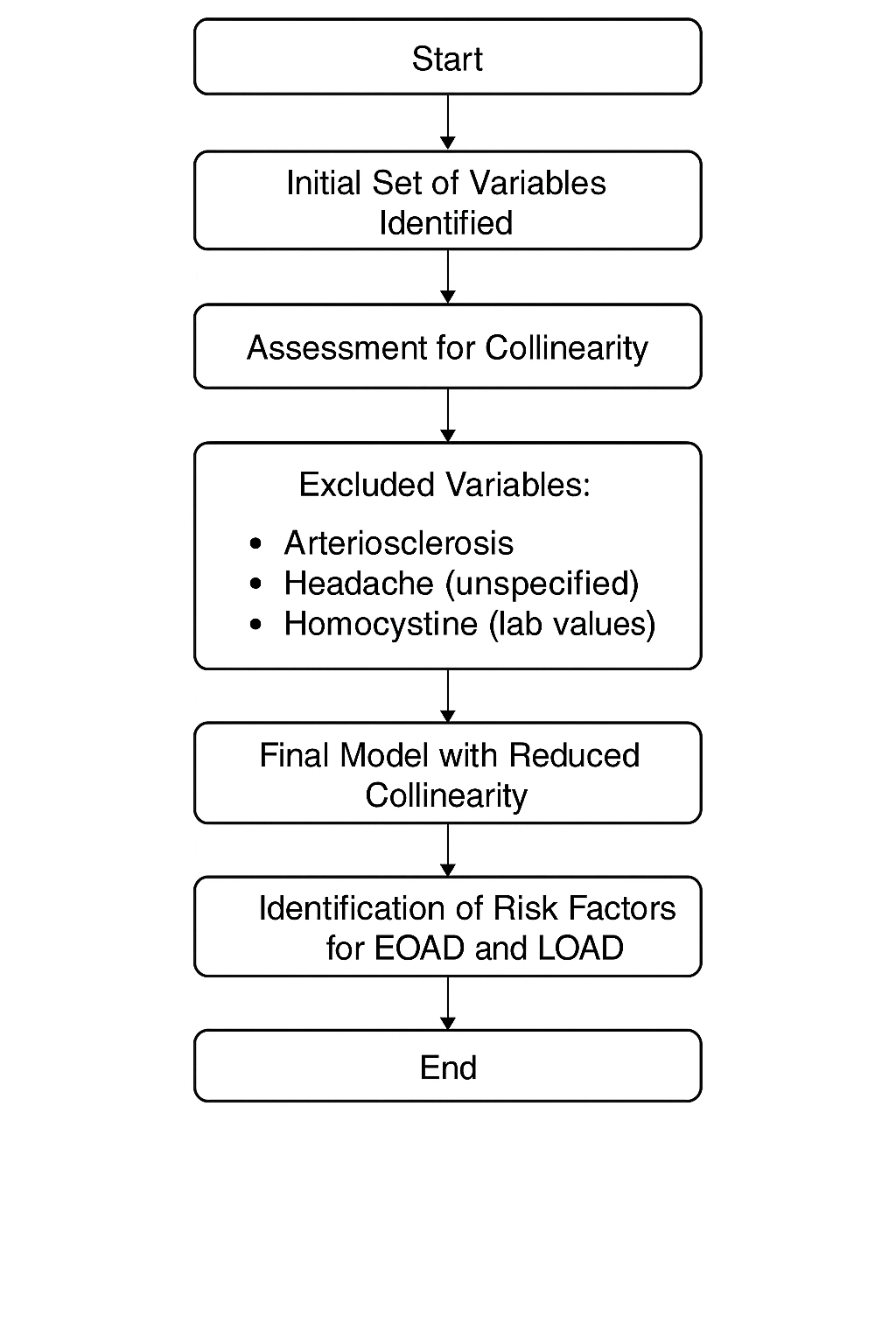
Supplement Figure 2. Flowchart for variable Selection Process to Identify Risk Factors in EOAD and LOAD patients stratified by sex, and variables excluded from the model.
